# A New Variational Bayesian Adaptive Extended Kalman Filter for Cooperative Navigation

**DOI:** 10.3390/s18082538

**Published:** 2018-08-03

**Authors:** Chengjiao Sun, Yonggang Zhang, Guoqing Wang, Wei Gao

**Affiliations:** 1 College of Automation, Harbin Engineering University, Harbin 150001, China; jiao_9128@163.com (C.S.); wangguoqing2014@hrbeu.edu.cn (G.W.); 2 School of Electrical Engineering and Automation, Harbin Institute of Technology, Harbin 150001, China; gaow@hit.edu.cn

**Keywords:** extended Kalman filter (EKF), variational Bayesian, cooperative navigation, nonlinear filters

## Abstract

To solve the problem of unknown state noises and uncertain measurement noises inherent in underwater cooperative navigation, a new Variational Bayesian (VB)-based Adaptive Extended Kalman Filter (VBAEKF) for master–slave Autonomous Underwater Vehicles (AUV) is proposed in this paper. The Inverse Wishart (IW) distribution is used to model the predicted error covariance and measurement noise covariance matrix. The state, together with the predicted error covariance and measurement noise covariance matrix, can be adaptively estimated based on VB approximation. The performance of the proposed algorithm is demonstrated through a lake trial, which shows the advantage of the proposed algorithm.

## 1. Introduction

With the continuous expansion of marine exploitations and the increasing complexity of military requirements, it will be difficult for a single Autonomous Underwater Vehicle (AUV) to achieve the desired goals. Therefore, a multi-AUV cooperative system has gradually become more popular [1,2,3]. Similar to a single AUV, the multi-AUV cooperative operation also requires accurate navigation and localization abilities [4,5]. In the master–slave cooperative navigation, it is generally considered that the AUVs with strong positioning ability are the master AUVs, and the master AUVs are the core of the AUV group that each slave AUV needs to communicate with. This cooperative navigation method has many advantages, such as low cost, easy realization, group resolution and flexible combination [6,7]. We aim to improve the navigation of slave AUVs by the filtering method, using the accurate location information of the master AUV and the relative observation between the master and slave AUVs.

The sensor of the multi-AUV cooperative navigation system includes various navigation, communication, and detection sensors, and they are the main sources of information for cooperative navigation. According to the object classification described by the sensor information, the navigation equipment can be divided into internal sensors and external sensors [8,9,10]. The internal sensors refer to the sensors used to measure the AUV’s own motion parameters, including Doppler Velocity Log (DVL), magnetic compass, and depth sensor. The DVL can measure the speed of the vehicle relative to the seabed, the magnetic compass is used to measure the vehicle heading information, while the pressure depth gauge can obtain the depth information. The external sensors include sensors for realizing underwater sound detection and underwater acoustic communication. In practical cooperative navigation, these sensors are affected by many factors such as temperature, salinity, depth, current, interface reflection and refraction in the underwater environment [11,12,13,14]. The underwater acoustic channel becomes a high noise channel with strong reverberation, narrow channel bandwidth, and multipath effects. The underwater acoustic communication of an AUV consisting of multiple sensors is constrained by these factors, making the noise statistical characteristics of the sensor inaccurate and even able to change over time. At this point, if the information fusion filter adopts the wrong noise covariance matrix such as the method described in [3], its accuracy may degrade or even diverge. Therefore, if real-time online estimation of the unknown process and measurement noise statistics can be performed in the filtering process, the filtered noise covariance matrix will act as an adaptive adjustment to meet the actual noise characteristics of the system, which will further improve the accuracy of AUV cooperative navigation [15,16].

In recent years, various adaptive filters have been proposed in order to solve the problems existing in conventional filters when the noise statistics are unknown or time-varying [12,17]. Based on maximum posterior estimation, the Sage-Husa adaptive Kalman filter can estimate the statistical characteristics of the system measurement noise in real time to improve the estimation accuracy of the filtering [18]. However, it requires that the system noise covariance and measurement noise covariance must be positive or positive definite, otherwise it will easily lead to filter divergence [19,20]. Fading adaptive filtering adjusts the weight of the newly measured data by increasing the one step predicted covariance matrix, but the calculation process of the scalar fade-in factor is cumbersome and has the same adjustment ability for each filter channel [21]. Although the maximum likelihood based adaptive filtering method can estimate and correct the second-order moments of the statistical characteristics of noise online, it needs to rely on an accurate innovation covariance estimate [22,23,24]. The existing Variational Baysian (VB) based adaptive Kalman filter can estimate an inaccurate and slowly varying measurement noise covariance matrix for linear Gaussian state-space models offline [17,25]. However, it is not suitable for the cooperative navigation model since it is nonlinear.

In this paper, in order to further improve the estimation accuracy of the filter when the noise statistical information is unknown or time-varying in AUV cooperative navigation, a Gaussian approximate extended Kalman filter based on VB is proposed. In order to solve the inaccurate measurement noise generated by underwater acoustic communication, and uncertain process noise during dead reckoning, this paper models the Inverse Wishart (IW) prior to the prediction error covariance matrix and measurement noise covariance matrix. This VB method is then used to calculate the system state together with the unknown noise parameters. The VB method is used to estimate the predicted error covariance matrix instead of process noise parameters, so that state estimation considers not only the change of noise, but also the variation of the predicted error covariance. The effectiveness of the proposed filtering algorithm was evaluated by using postprocessed data collected in lake trials, which shows that the proposed filter has significantly improved robustness of unknown or time-varying noises than existing filters.

The main structure is listed as follows. In Section 2, we provide a short review of the cooperative navigation model using acoustic range measurement. In Section 3, a new Variational Bayesian-based Adaptive Extended Kalman Filter (VBAEKF) is derived. In Section 4, the proposed algorithm and existing state-of-the-art algorithms are tested in the actual lake field trial to show the excellent performance of the proposed algorithm. Finally, Section 5 discusses our conclusions.

## 2. System Model

To achieve master–slave cooperative navigation, master AUVs are usually equipped with a high-precision Inertial Navigation System (INS), DVL, and depth sensors to form a high-precision integrated navigation system for underwater navigation. Another option is having the master AUVs periodically emerge to receive high-precision GPS location information to achieve its own high-precision navigation and positioning. The slave AUV only has a low-precision magnetic compass, DVL, and depth sensors to perform dead reckoning. During the operation of the system, when the slave AUV uses an underwater acoustic communication device to obtain high-precision AUV position information and relative range reference information from the master AUV, this reference information can be used to realize the self-correction of its own position error and realize the cooperative navigation between vehicles [3,7].

Since the actual depth information can be directly measured by the pressure sensor in real time with bounded error, the depth does not need to be considered in the process equations and the practical working environment of the slave AUV can be simplified to a two-dimensional (2D) space. When calculating the relative range between the AUVs, the depth information is required. If the initial position $X_{1}={\left[x_{1},x_{2}\right]}^{T}$ of the slave AUV is known, the position at time k will be calculated in real time according to the speed and azimuth information measured by its own sensors as follows:

(1) $$\left\{ \begin{array}{l} x_{k}=x_{k-1}+\Delta t\left({\hat{v}}_{k}\cos{\hat{\theta }}_{k}+{\hat{w}}_{k}\sin{\hat{\theta }}_{k}\right)+\omega_{x,k-1}\\ y_{k}=y_{k-1}+\Delta t\left({\hat{v}}_{k}\sin{\hat{\theta }}_{k}-{\hat{w}}_{k}\cos{\hat{\theta }}_{k}\right)+\omega_{y,k-1} \end{array}\right.,$$

where $x_{k}$ and $y_{k}$ respectively denote the eastward and northward position of the AUV at time k, Δt is the sampling interval, ${\hat{v}}_{k}$ and ${\hat{w}}_{k}$ denote the forward and starboard velocity measurement information of the DVL along the vehicle coordinate system, and ${\hat{\theta }}_{k}$ is the heading measured by the magnetic compass. $\omega_{k}={\left[\omega_{x,k},\omega_{y,k}\right]}^{T}$ is system process noise, including speed measurement noise and azimuth measurement noise.

The slave AUV uses the relative range measurement to correct the navigation error. Therefore, the measurement equation is the range between the master AUV and slave AUV, which can be written as follows:

(2) $$Z_{k}=\sqrt{{\left(x_{k}-x_{k}^{m}\right)}^{2}+{\left(y_{k}-y_{k}^{m}\right)}^{2}}+\upsilon_{k},$$

where $X_{k}^{m}={\left[x_{k}^{m},y_{k}^{m}\right]}^{T}$ and $X_{k}={\left[x_{k},y_{k}\right]}^{T}$ represent the position of the master AUV and the slave AUV at time step k, respectively. In addition, $\upsilon_{k}$ is the measurement noise.

According to the dynamic system model (1) and measurement model (2), the discrete-time state space equation from the AUV can be written as:

(3) $$X_{k}=FX_{k-1}+\mu_{k}+\omega_{k-1},$$

(4) $$Z_{k}=h(X_{k},x_{k}^{m},y_{k}^{m})+\upsilon_{k},$$

where the state transition matrix $F=I_{2}$ and $I_{2}$ represent a two-dimensional identity matrix. The control input is $\mu_{k}={\left[\Delta t\left({\hat{v}}_{k}\cos{\hat{\theta }}_{k}+{\hat{w}}_{k}\sin{\hat{\theta }}_{k}\right),\Delta t\left({\hat{v}}_{k}\sin{\hat{\theta }}_{k}-{\hat{w}}_{k}\cos{\hat{\theta }}_{k}\right)\right]}^{T}$ and $h(X_{k},x_{k}^{m},y_{k}^{m})=\sqrt{{\left(x_{k}-x_{k}^{m}\right)}^{2}+{\left(y_{k}-y_{k}^{m}\right)}^{2}}$. $\omega_{k}$ and $\upsilon_{k}$ are system process noise and measurement noise, respectively, and are usually assumed to be independent zero-mean Gaussian white noise sequences that satisfy the distributions $\omega_{k}∼N(0,Q_{k})$ and $\upsilon_{k}∼N\left(0,R_{k}\right)$. The measurement Equation (4) shows that h(⋅) is non-linear, so it is necessary to apply the partial derivative matrix to the proposed algorithm. The partial derivative matrix of the measurement equation can be written as:

(5) $$\begin{array}{ll} H_{k} & =\frac{\partial h(X_{k},x_{k}^{m},y_{k}^{m})}{\partial X_{k}}{|}_{X_{k}={\hat{X}}_{k|k-1}}\\ & =\left[\frac{{\hat{x}}_{k|k-1}-x_{k}^{m}}{\sqrt{{\left({\hat{x}}_{k|k-1}-x_{k}^{m}\right)}^{2}+{\left({\hat{y}}_{k|k-1}-y_{k}^{m}\right)}^{2}}},\frac{{\hat{y}}_{k|k-1}-y_{k}^{m}}{\sqrt{{\left({\hat{x}}_{k|k-1}-x_{k}^{m}\right)}^{2}+{\left({\hat{y}}_{k|k-1}-y_{k}^{m}\right)}^{2}}}\right] \end{array},$$

where ${\hat{X}}_{k|k-1}={\left[{\hat{x}}_{k|k-1},{\hat{y}}_{k|k-1}\right]}^{T}$ represents k moment predicted position of the slave AUV.

## 3. The Proposed Cooperative Navigation Algorithm Based on Variational Bayesian

The previous section studied the multi-AUV’s cooperative navigation technology under ideal communication; that is, suppose we know the exact mathematical model of the system, and both the system noise and the measurement noise satisfy the Gaussian distribution. However, during the actual navigation of AUVs, the underwater environment is affected by factors such as temperature, season, and current layer, while the underwater acoustic distance observation is often interfered with by abnormal measurement noise. The resulting measurement noises and its covariance matrix are often unknown or inaccurate. At the same time, the process equation of the system is influenced by the dead reckoning sensor, and the process noise often cannot be a constant value. The variational Bayesian method is introduced to solve this problem [17,26].

In the conventional Kalman filtering model, the one-step predicted Probability Density Function (PDF) $p(X_{k}|Z_{1:k-1})$ and likelihood PDF $p\left(Z_{k}|X_{k}\right)$ are normal distributions:

(6) $$p(X_{k}|Z_{1:k-1})=N(X_{k};{\hat{X}}_{k|k-1},P_{k|k-1}),$$

(7) $$p\left(Z_{k}|X_{k}\right)=N(Z_{k};h(X_{k}),R_{k}),$$

where N(A;γ,Σ) is the normal distribution with mean γ and variance Σ, and the probability density function of the normal distribution is:

(8) $$N(A;\gamma ,\Sigma )=\frac{1}{\sqrt{\left|2\pi \Sigma \right|}}e^{-\frac{1}{2}{\left(A-\gamma \right)}^{T}\Sigma^{-1}(A-\gamma )}.$$

According to Equation (3), the predicted state vector ${\hat{X}}_{k|k-1}$ and the corresponding one-step predicted covariance matrix $P_{k|k-1}$ can be written as:

(9) $${\hat{X}}_{k|k-1}=F{\hat{X}}_{k-1|k-1}+\mu_{k},$$

(10) $$P_{k|k-1}=FP_{k-1|k-1}F^{T}+Q_{k-1},$$

where ${\hat{X}}_{k-1|k-1}$ and $P_{k-1|k-1}$ respectively represent the state estimation at time k−1 and the corresponding estimation error covariance matrix. Note that the $P_{k|k-1}$ in Equation (10) is not exact, because the true process noise covariance matrix $Q_{k}$ is unknown due to the effects of complex underwater environments.

In Bayesian statistics, the Inverse Wishart (IW) distribution can be viewed as the conjugate prior distribution for the covariance matrix of a normal distribution with known mean [27]. If the inverse matrix $B^{-1}$ of a positive definite matrix B follows the Wishart distribution $W(B^{-1};\lambda ,\Psi^{-1})$, then the matrix B follows the IW distribution:

(11) $$IW(B;\lambda ,\Psi )=\frac{{\left|\Psi \right|}^{\lambda /2}{\left|B\right|}^{-(\lambda +d+1)/2}e^{-trace(\Psi B^{-1})/2}}{2^{d\lambda /2}\Gamma_{d}(\lambda /2)},$$

where λ>d+1 is the degree of freedom, Ψ is a d×d positive definite matrix, d is the dimension of Ψ, $\Gamma_{d}(\cdot )$ is the multivariate gamma distribution, and trace(⋅) is the trace function. If the matrix B∼IW(B;λ,Ψ) obeys the IW distribution, its expectation is [27]:

(12) $$E(B)=\frac{\Psi }{\lambda -d-1}.$$

Since $P_{k|k-1}$ and $R_{k}$ are the covariance matrices of the normal PDFs, their prior distributions $p(P_{k|k-1}|{Ζ}_{1:k-1})$ and $p(R_{k}|{Ζ}_{1:k-1})$ can be written as IW distributions:

(13) $$p(P_{k|k-1}|{Ζ}_{1:k-1})=IW(P_{k|k-1};{\hat{t}}_{k|k-1},{\hat{T}}_{k|k-1}),$$

(14) $$p(R_{k}|{Ζ}_{1:k-1})=IW(R_{k|k-1};{\hat{u}}_{k|k-1},{\hat{U}}_{k|k-1}),$$

where ${\hat{t}}_{k|k-1}$ and ${\hat{T}}_{k|k-1}$ represent the degrees of freedom and scale matrix of $p(P_{k|k-1}|{Ζ}_{1:k-1})$, ${\hat{u}}_{k|k-1}$ and ${\hat{U}}_{k|k-1}$ represent the degrees of freedom and scale matrix of $p(R_{k}|{Ζ}_{1:k-1})$, respectively. Next, we need to get the value of ${\hat{t}}_{k|k-1}$, ${\hat{T}}_{k|k-1}$, ${\hat{u}}_{k|k-1}$, and ${\hat{U}}_{k|k-1}$.

The mean value of $P_{k|k-1}$ can be set as the nominal predicted error covariance matrix ${\bar{P}}_{k|k-1}$. According to Equation (12), we obtain:

(15) $$\frac{{\hat{T}}_{k|k-1}}{{\hat{t}}_{k|k-1}-n-1}={\bar{P}}_{k|k-1}=FP_{k-1|k-1}F^{T}+{\bar{Q}}_{k-1},$$

where n represents the dimension of ${\hat{T}}_{k|k-1}$ the state and ${\bar{Q}}_{k-1}$ represents the nominal process noise covariance. Let:

(16) $${\hat{t}}_{k|k-1}=n+\tau +1,$$

where τ≥0 is a tuning parameter.

Substituting (16) into (15) gives:

(17) $${\hat{T}}_{k|k-1}=\tau {\bar{P}}_{k|k-1}.$$

According to Bayes’ theorem, prior distribution $p(R_{k}|{Ζ}_{1:k-1})$ can be written as [17]:

(18) $$p(R_{k}|{Ζ}_{1:k-1})=\int p(R_{k}|R_{k-1})p(R_{k-1}|{Ζ}_{1:k-1})dR_{k-1},$$

where $p(R_{k-1}|{Ζ}_{1:k-1})$ is the posterior probability density function of the measurement noise covariance matrix $R_{k-1}$.

According to Equation (14), the prior distribution $p(R_{k-1}|{Ζ}_{1:k-2})$ of the measurement noise covariance matrix $R_{k-1}$ is subject to the IW distribution, and its posterior distribution $p(R_{k-1}|{Ζ}_{1:k-1})$ should also be an IW distribution as follows:

(19) $$p(R_{k-1}|{Ζ}_{1:k-1})=IW(R_{k-1};{\hat{u}}_{k-1|k-1},{\hat{U}}_{k-1|k-1}).$$

In practical applications, the dynamic model $p(R_{k}|R_{k-1})$ of the noise variance of Equation (18) is unknown. So we choose a factor ρ to preserve and propagate the approximate posterior at the previous moment [28], and the prior parameters can be written as:

(20) $${\hat{u}}_{k|k-1}=\rho ({\hat{u}}_{k-1|k-1}-m-1)+m+1,$$

(21) $${\hat{U}}_{k|k-1}=\rho {\hat{U}}_{k-1|k-1},$$

where ρ∈(0,1] is a forgetting factor, which represents the degree of fluctuation over time. ρ=1 represents the steady state variance; the smaller the value of ρ, the greater the frequency of fluctuations over time.

In order to estimate the state $X_{k}$, the predicted state error covariance $P_{k|k-1}$ and the measurement noise covariance matrix $R_{k}$, we need to calculate their joint posterior probability density function $p(X_{k},P_{k|k-1},R_{k}|Z_{1:k})$. We use the variational Bayesian method and find an approximate PDF of a free form as follows [28,29]:

(22) $$p(X_{k},P_{k|k-1},R_{k}|Z_{1:k})\approx q(X_{k})q(P_{k|k-1})q(R_{k}),$$

where q(⋅) denotes the approximate posterior PDF of p(⋅). The VB-approximation can now be formed by minimizing the Kullback-Leibler Divergence (KLD) between the approximation posterior PDF $q(X_{k})$, $q(P_{k|k-1})$, $q(R_{k})$ and the true joint posterior $p(X_{k},P_{k|k-1},R_{k}|Z_{1:k})$ [29,30]:

(23) $$\left\{q(X_{k}),q(P_{k|k-1}),q(R_{k})\right\}=\arg\;\min\;KLD\left(q(X_{k}),q(P_{k|k-1}),q(R_{k})∥p(X_{k},P_{k|k-1},R_{k}|Z_{1:k})\right),$$

where *KLD* represents relative entropy, which is defined as follows:

(24) $$KLD(q(X)∥p(X))=\int q(X)\log\frac{q(X)}{p(X)}dX.$$

The optimal solution of Equation (22) satisfies the following equation [29]:

(25) $$\log q(X_{k})=E_{P_{k|k-1},R_{k}}\left[\log p(X_{k},P_{k|k-1},R_{k},Z_{1:k})\right]+c_{X_{k}},$$

(26) $$\log q(P_{k|k-1})=E_{X_{k},R_{k}}\left[\log p(X_{k},P_{k|k-1},R_{k},Z_{1:k})\right]+c_{P_{k|k-1}},$$

(27) $$\log q(R_{k})=E_{X_{k},P_{k|k-1}}\left[\log p(X_{k},P_{k|k-1},R_{k},Z_{1:k})\right]+c_{R_{x}},$$

where $c_{X_{k}}$, $c_{P_{k|k-1}}$, $c_{R_{x}}$ represent the constants with respect to variable $X_{k}$, predicted error covariance $P_{k|k-1}$ and measurement noise covariance matrix $R_{k}$, respectively. log(⋅) represents the logarithm function, and $E_{\Theta }\left[\cdot \right]$ represents the expectation of the approximate posterior PDF of the variable Θ. Because $q(X_{k})$, $q(P_{k|k-1})$, $q(R_{k})$ are coupled, Equations (25)–(27) cannot be directly solved. These parameters can be solved by the fixed-point iterative method.

Therefore, (26) can be further written as (see the Appendix A for a detail derivation):

(28) $$\log q^{\left(i+1\right)}(P_{k|k-1})=-\frac{1}{2}(n+{\hat{t}}_{k|k-1}+2)\log\left|P_{k|k-1}\right|-\frac{1}{2}trace\left(\left(A_{k}^{\left(i\right)}+{\hat{T}}_{k|k-1}\right)P_{k|k-1}^{-1}\right)+c_{P_{k|k-1}},$$

where $q^{\left(i+1\right)}(\cdot )$ represents the approximate PDF of the i+1th iteration of q(⋅), and $A_{k}^{\left(i\right)}$ is defined as:

(29) $$\begin{array}{cc} A_{k}^{\left(i\right)} & =E^{\left(i\right)}\left[(X_{k}-{\hat{X}}_{k|k-1}){\left(X_{k}-{\hat{X}}_{k|k-1}\right)}^{T}\right]\\ & =E^{\left(i\right)}\left[(X_{k}-{\hat{X}}_{k|k}^{\left(i\right)}+{\hat{X}}_{k|k}^{\left(i\right)}-{\hat{X}}_{k|k-1}){\left(X_{k}-{\hat{X}}_{k|k}^{\left(i\right)}+{\hat{X}}_{k|k}^{\left(i\right)}-{\hat{X}}_{k|k-1}\right)}^{T}\right]\\ & =E^{\left(i\right)}\left[(X_{k}-{\hat{X}}_{k|k}^{\left(i\right)}){\left(X_{k}-{\hat{X}}_{k|k}^{\left(i\right)}\right)}^{T}\right]+({\hat{X}}_{k|k}^{\left(i\right)}-{\hat{X}}_{k|k-1}){\left({\hat{X}}_{k|k}^{\left(i\right)}-{\hat{X}}_{k|k-1}\right)}^{T}\\ & =P_{k|k}^{\left(i\right)}+({\hat{X}}_{k|k}^{\left(i\right)}-{\hat{X}}_{k|k-1}){\left({\hat{X}}_{k|k}^{\left(i\right)}-{\hat{X}}_{k|k-1}\right)}^{T}, \end{array}$$

where $E^{\left(i\right)}\left[\rho \right]$ represents the expectation of variable ρ at the i-th iteration.

According to (28), $q^{\left(i+1\right)}(P_{k|k-1})$ can be viewed as the IW distribution with the degree of freedom ${\hat{t}}_{k|k-1}^{\left(i+1\right)}$ and scale matrix ${\hat{T}}_{k|k-1}^{\left(i+1\right)}$:

(30) $$q^{\left(i+1\right)}(P_{k|k-1})=IW(P_{k|k-1};{\hat{t}}_{k}^{\left(i+1\right)},{\hat{T}}_{k}^{\left(i+1\right)}),$$

where the degree of freedom ${\hat{t}}_{k|k-1}^{\left(i+1\right)}$ and scale matrix ${\hat{T}}_{k|k-1}^{\left(i+1\right)}$ can be expressed as

(31) $${\hat{t}}_{k}^{\left(i+1\right)}={\hat{t}}_{k|k-1}+1,$$

(32) $${\hat{T}}_{k}^{\left(i+1\right)}=A_{k}^{\left(i\right)}+{\hat{T}}_{k|k-1}.$$

Equation (27) can be further written as:

(33) $$\log q^{\left(i+1\right)}(R_{k})=-\frac{1}{2}(m+{\hat{u}}_{k|k-1}+2)\log\left|R_{k}\right|-\frac{1}{2}trace\left(\left(B_{k}^{\left(i\right)}+{\hat{U}}_{k|k-1}\right)R_{k}^{-1}\right)+c_{R_{k}},$$

where $B_{k}^{\left(i\right)}$ is defined as:

(34) $$\begin{array}{cc} B_{k}^{\left(i\right)} & =E^{i}\left[(Z_{k}-h(X_{k})){\left(Z_{k}-h(X_{k})\right)}^{T}\right]\\ & =\int \left(Z_{k}-h(X_{k})){\left(Z_{k}-h(X_{k})\right)}^{T}N(X_{k};{\hat{X}}_{k|k}^{\left(i\right)},P_{k|k}^{\left(i\right)}\right)dX_{k}. \end{array}$$

According to (33), $q^{\left(i+1\right)}(R_{k})$ can be viewed as the IW distribution with the degree of freedom ${\hat{u}}_{k|k-1}^{\left(i+1\right)}$ and scale matrix ${\hat{U}}_{k|k-1}^{\left(i+1\right)}$

(35) $$q^{\left(i+1\right)}(R_{k})=IW(R_{k};{\hat{u}}_{k}^{\left(i+1\right)},{\hat{U}}_{k}^{\left(i+1\right)}),$$

where the degree of freedom ${\hat{u}}_{k|k-1}^{\left(i+1\right)}$ and scale matrix ${\hat{U}}_{k|k-1}^{\left(i+1\right)}$ can be expressed as

(36) $${\hat{u}}_{k}^{\left(i+1\right)}={\hat{u}}_{k|k-1}+1,$$

(37) $${\hat{U}}_{k}^{\left(i+1\right)}=B_{k}^{\left(i\right)}+{\hat{U}}_{k|k-1}.$$

Equation (25) can be further written as:

(38) $$\begin{array}{cc} \log q^{\left(i+1\right)}(X_{k}) & =-\frac{1}{2}{\left(Z_{k}-h(X_{k})\right)}^{T}E^{\left(i+1\right)}\left[R_{k}^{-1}\right]\left(Z_{k}-h(X_{k})\right)\\ & -\frac{1}{2}{\left(X_{k}-{\hat{X}}_{k|k-1}\right)}^{T}E^{\left(i+1\right)}\left[P_{k|k-1}^{-1}\right](X_{k}-{\hat{X}}_{k|k-1})+c_{X_{k}}, \end{array}$$

where $E^{\left(i+1\right)}\left[R_{k}^{-1}\right]$ and $E^{\left(i+1\right)}\left[P_{k|k-1}^{-1}\right]$ can be represented as the following equation [29]:

(39) $$E^{\left(i+1\right)}\left[R_{k}^{-1}\right]=({\hat{u}}_{k}^{\left(i+1\right)}-m-1){\left({\hat{U}}_{k}^{\left(i+1\right)}\right)}^{-1},$$

(40) $$E^{\left(i+1\right)}\left[P_{k|k-1}^{-1}\right]=({\hat{t}}_{k}^{\left(i+1\right)}-n-1){\left({\hat{T}}_{k}^{\left(i+1\right)}\right)}^{-1}.$$

The one-step predicted PDF $p^{\left(i+1\right)}(X_{k}|Z_{1:k-1})$ and likelihood PDF $p\left(Z_{k}|X_{k}\right)$ after updating the i+1th iteration can be written in the following equations:

(41) $$p^{\left(i+1\right)}(X_{k}|Z_{1:k-1})=N(X_{k};{\hat{X}}_{k|k-1},{\hat{P}}_{k|k-1}^{\left(i+1\right)}),$$

(42) $$p^{\left(i+1\right)}\left(Z_{k}|X_{k}\right)=N\left(Z_{k};h(X_{k}),{\hat{R}}_{k}^{\left(i+1\right)}\right).$$

The corrected predicted error covariance matrix ${\hat{P}}_{k|k-1}^{\left(i+1\right)}$ and the measurement noise covariance matrix ${\hat{R}}_{k}^{\left(i+1\right)}$ can be written as:

(43) $${\hat{P}}_{k|k-1}^{\left(i+1\right)}={\left\{E^{\left(i+1\right)}\left[P_{k|k-1}^{-1}\right]\right\}}^{-1},$$

(44) $${\hat{R}}_{k}^{\left(i+1\right)}={\left\{E^{\left(i+1\right)}\left[R_{k}^{-1}\right]\right\}}^{-1}.$$

Employing (41)–(44) in (38), we have:

(45) $$q^{\left(i+1\right)}(X_{k})=\frac{1}{c_{k}^{\left(i+1\right)}}p^{\left(i+1\right)}\left(Z_{k}|X_{k}\right)p^{\left(i+1\right)}(X_{k}|Z_{1:k-1}),$$

where the normalization constant $c_{k}^{\left(i+1\right)}$ is given by:

(46) $$c_{k}^{\left(i+1\right)}=\int p^{\left(i+1\right)}\left(Z_{k}|X_{k}\right)p^{\left(i+1\right)}(X_{k}|Z_{1:k-1})dX_{k}.$$

Considering (41)–(46), it can be upgraded to a Gaussian distribution with mean $X_{k|k}^{\left(i+1\right)}$ and variance $P_{k|k}^{\left(i+1\right)}$:

(47) $$q^{\left(i+1\right)}(X_{k})=N\left(X_{k};X_{k|k}^{\left(i+1\right)},P_{k|k}^{\left(i+1\right)}\right),$$

where mean $X_{k|k}^{\left(i+1\right)}$ and variance $P_{k|k}^{\left(i+1\right)}$ at i+1th iteration are calculated as:

(48) $$K_{k}^{\left(i+1\right)}={\hat{P}}_{k|k-1}^{\left(i+1\right)}H_{k}^{T}{\left(H_{k}{\hat{P}}_{k|k-1}^{\left(i+1\right)}H_{k}^{T}+{\hat{R}}_{k}^{\left(i+1\right)}\right)}^{-1},$$

(49) $${\hat{X}}_{k}^{\left(i+1\right)}={\hat{X}}_{k|k-1}+K_{k}^{\left(i+1\right)}\left(Z_{k}-h({\hat{X}}_{k|k-1})\right),$$

(50) $${\hat{P}}_{k|k}^{\left(i+1\right)}={\hat{P}}_{k|k-1}^{\left(i+1\right)}-K_{k}^{\left(i+1\right)}H_{k}{\hat{P}}_{k|k-1}^{\left(i+1\right)}.$$

After fixed-point iteration N, we obtain the variational approximation of the posterior PDFs:

(51) $$q(X_{k})\approx q^{\left(N\right)}(X_{k})=Ν(X_{k};{\hat{X}}_{k|k}^{\left(N\right)},P_{k|k}^{\left(N\right)})=Ν(X_{k};{\hat{X}}_{k|k},P_{k|k}),$$

(52) $$q(P_{k|k-1})\approx q^{\left(N\right)}(P_{k|k-1})=IW(P_{k|k-1};{\hat{t}}_{k}^{\left(N\right)},{\hat{T}}_{k}^{\left(N\right)})=IW(P_{k|k-1};{\hat{t}}_{k|k},{\hat{T}}_{k|k}),$$

(53) $$q(R_{k})\approx q^{\left(N\right)}(R_{k})=IW(R_{k};{\hat{u}}_{k}^{\left(N\right)},{\hat{U}}_{k}^{\left(N\right)})=IW(P_{k|k-1};{\hat{u}}_{k|k},{\hat{U}}_{k|k}).$$

The variational Bayesian adaptive EKF proposed in this paper consists of Equations (9), (15)–(17), (20), (21) and the measurement update process in Equations (29)–(32), (34)–(37), (39), (40), (43), (44), (47)–(53). The implementation pseudocode for the proposed adaptive cooperative navigation algorithm is shown in Algorithm 1.


**Algorithm 1: One-time step of the proposed VBAEKF for cooperative localization**

**Inputs:**
${\hat{X}}_{k-1|k-1},P_{k-1|k-1},{\hat{u}}_{k-1|k-1},{\hat{U}}_{k-1|k-1},F,x_{k}^{m},y_{k}^{m},h(X_{k},x_{k}^{m},y_{k}^{m}),Z_{k},{\bar{Q}}_{k-1},m,n,\tau ,\rho ,N$

**Time update:**

**1.**
${\hat{X}}_{k|k-1}=F{\hat{X}}_{k-1|k-1}+\mu_{k}$

**2.**
${\bar{P}}_{k|k-1}=FP_{k-1|k-1}F^{T}+{\bar{Q}}_{k-1}$

**Iterated measurement update:**

**3. Initialization:**

$\begin{array}{l} {\hat{X}}_{k|k}^{\left(0\right)}={\hat{X}}_{k|k-1},P_{k|k}^{\left(0\right)}={\bar{P}}_{k|k-1},{\hat{t}}_{k|k-1}=n+\tau +1,{\hat{T}}_{k|k-1}=\tau {\bar{P}}_{k|k-1},\\ {\hat{u}}_{k|k-1}=\rho \left({\hat{u}}_{k-1|k-1}-m-1\right)+m+1,{\hat{U}}_{k|k-1}=\rho {\hat{U}}_{k-1|k-1} \end{array}$
 **for**
i=0:N−1 **Update**
$q^{\left(i+1\right)}(P_{k|k-1})=IW(P_{k|k-1};{\hat{t}}_{k}^{\left(i+1\right)},{\hat{T}}_{k}^{\left(i+1\right)})$
**given**
$q^{\left(i\right)}(X_{k})$:
**4.**
$H_{k}=\left[\frac{{\hat{x}}_{k|k-1}-x_{k}^{m}}{\sqrt{{\left({\hat{x}}_{k|k-1}-x_{k}^{m}\right)}^{2}+{\left({\hat{y}}_{k|k-1}-y_{k}^{m}\right)}^{2}}},\frac{{\hat{y}}_{k|k-1}-y_{k}^{m}}{\sqrt{{\left({\hat{x}}_{k|k-1}-x_{k}^{m}\right)}^{2}+{\left({\hat{y}}_{k|k-1}-y_{k}^{m}\right)}^{2}}}\right]$

**5.**
$A_{k}^{\left(i\right)}=P_{k|k}^{\left(i\right)}+({\hat{X}}_{k|k}^{\left(i\right)}-{\hat{X}}_{k|k-1}){\left({\hat{X}}_{k|k}^{\left(i\right)}-{\hat{X}}_{k|k-1}\right)}^{T}$
**6.**${\hat{t}}_{k|k-1}^{\left(i+1\right)}={\hat{t}}_{k|k-1}+1$, ${\hat{T}}_{k|k-1}^{\left(i+1\right)}=A_{k}^{\left(i\right)}+{\hat{T}}_{k|k-1}$ **Update**
$q^{\left(i+1\right)}(R_{k})=IW(R_{k};{\hat{u}}_{k}^{\left(i+1\right)},{\hat{U}}_{k}^{\left(i+1\right)})$
**given**
$q^{\left(i\right)}(X_{k})$:
**7.**
$B_{k}^{\left(i\right)}=\left(Z_{k}-h(X_{k|k},x_{k}^{m},y_{k}^{m})\right){\left(Z_{k}-h(X_{k|k},x_{k}^{m},y_{k}^{m})\right)}^{T}+H_{k}P_{k|k}^{\left(i\right)}{\left(H_{k}\right)}^{T}$
**8.**${\hat{u}}_{k}^{\left(i+1\right)}={\hat{u}}_{k|k-1}+1$, ${\hat{U}}_{k}^{\left(i+1\right)}=B_{k}^{\left(i\right)}+{\hat{U}}_{k|k-1}$**Update**$q^{\left(i+1\right)}(X_{k})=N\left(X_{k};X_{k|k}^{\left(i+1\right)},P_{k|k}^{\left(i+1\right)}\right)$**given**$q^{\left(i+1\right)}(P_{k|k-1})$**and**$q^{\left(i+1\right)}(R_{k})$:**9.**$E^{\left(i+1\right)}\left[R_{k}^{-1}\right]=({\hat{u}}_{k}^{i+1}-m-1){\left({\hat{U}}_{k}^{i+1}\right)}^{-1}$, $E^{\left(i+1\right)}\left[P_{k|k-1}^{-1}\right]=({\hat{t}}_{k}^{i+1}-n-1){\left({\hat{T}}_{k}^{i+1}\right)}^{-1}$**10.**${\hat{P}}_{k|k-1}^{\left(i+1\right)}={\left\{E^{\left(i+1\right)}\left[P_{k|k-1}^{-1}\right]\right\}}^{-1}$, ${\hat{R}}_{k}^{\left(i+1\right)}={\left\{E^{\left(i+1\right)}\left[R_{k}^{-1}\right]\right\}}^{-1}$
**11.**
$K_{k}^{\left(i+1\right)}={\hat{P}}_{k|k-1}^{\left(i+1\right)}{\left(H_{k}\right)}^{T}{\left(H_{k}{\hat{P}}_{k|k-1}^{\left(i+1\right)}{\left(H_{k}\right)}^{T}+{\hat{R}}_{k}^{\left(i+1\right)}\right)}^{-1}$

**12.**
${\hat{X}}_{k}^{\left(i+1\right)}={\hat{X}}_{k|k-1}+K_{k}^{\left(i+1\right)}\left(Z_{k}-h({\hat{X}}_{k|k-1},x_{k}^{m},y_{k}^{m})\right)$

**13.**
${\hat{P}}_{k|k}^{\left(i+1\right)}={\hat{P}}_{k|k-1}^{\left(i+1\right)}-K_{k}^{\left(i+1\right)}H_{k}{\hat{P}}_{k|k-1}^{\left(i+1\right)}$
  **end for**
**14.**
${\hat{X}}_{k|k}={\hat{X}}_{k|k}^{\left(N\right)},P_{k|k}=P_{k|k}^{\left(N\right)},{\hat{t}}_{k|k}={\hat{t}}_{k}^{\left(N\right)},{\hat{T}}_{k|k}={\hat{T}}_{k}^{\left(N\right)},{\hat{u}}_{k|k}={\hat{u}}_{k}^{\left(N\right)},{\hat{U}}_{k|k}={\hat{U}}_{k}^{\left(N\right)}$
  **Outputs:**
${\hat{X}}_{k|k},P_{k|k},{\hat{t}}_{k|k},{\hat{T}}_{k|k},{\hat{u}}_{k|k},{\hat{U}}_{k|k}$

The tuning parameter τ is a key parameter to implement the proposed VBAEKF. In general, τ is deemed as a harmonic weight to balance the efficacy of the nominal predicted error covariance matrix ${\bar{P}}_{k|k-1}$ and $A_{k}^{\left(i\right)}$, a smaller tuning parameter means that a large quantity of information about the process model is lost. On the other hand, when τ becomes larger, the substantial prior uncertainties induced by the inaccurate nominal predicted error covariance matrix will factor into the measurement update. In this paper, the tuning parameter is selected as τ=2.

## 4. Field Trial Results

**Figure 1:**
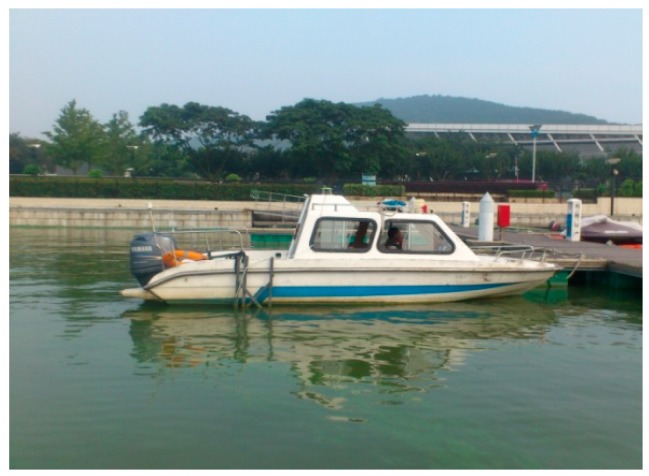
The vessel employed in the experiment.

**Figure 2:**
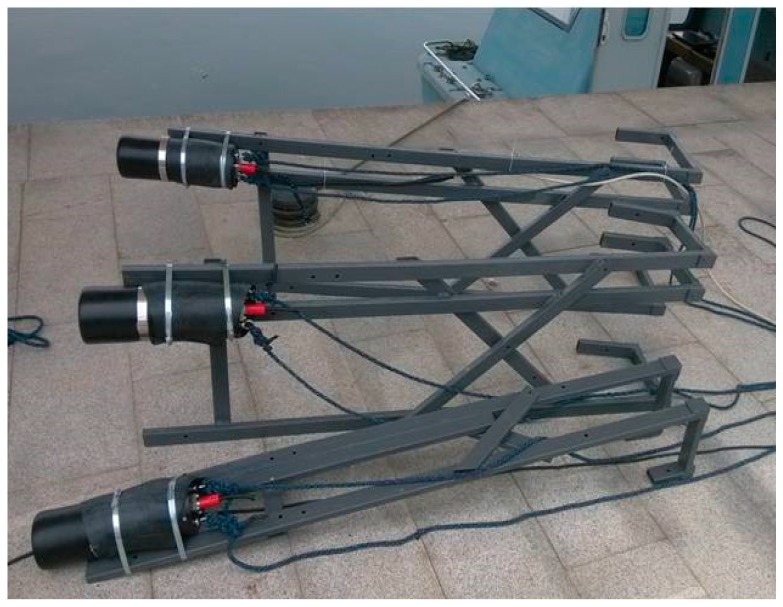
S2CR 7/17 acoustic equipment.

**Figure 3:**
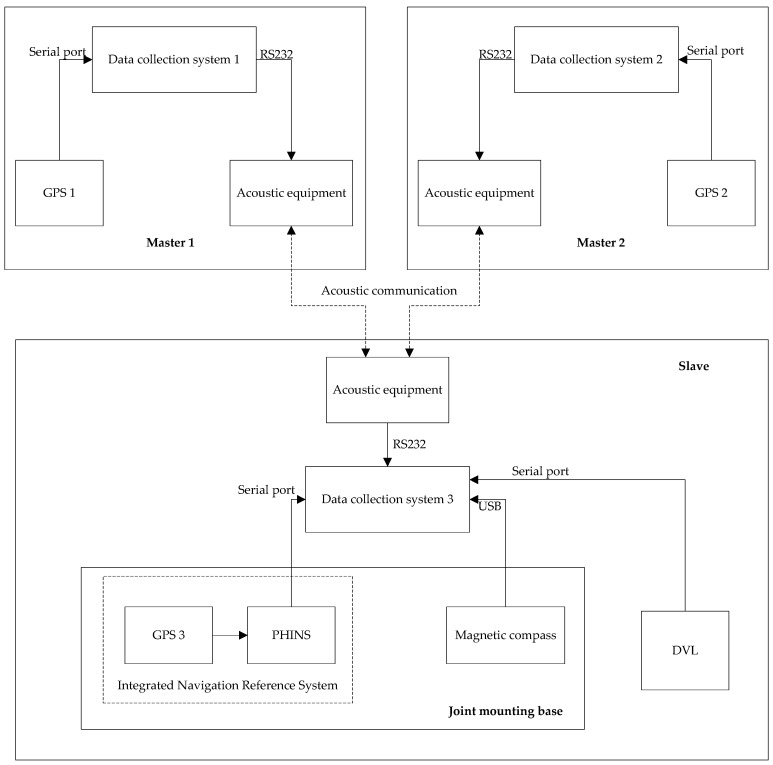
Experiment scheme of cooperative navigation. DVL: Doppler Velocity Log; PHINS: Photonics Inertial Navigation System.

The effectiveness and superiority of the adaptive cooperative navigation algorithm proposed in this paper is validated in practical systems by using the offline experimental data collected in a lake trial experiment. This experiment was conducted in August 2014 in Taihu Lake, Wuxi. Subject to experimental conditions, the lake test was carried out using the surface boat and acoustic communication equipment shown in Figure 1 and Figure 2. The experimental constitution scheme of the cooperative navigation lake test is shown in Figure 3. Three survey vessels are used in the test. Two vessels acted as the master AUVs and the other is the slave AUV. Each AUV is equipped with underwater acoustic communication equipment S2CR 7/17 (Evologics, Berlin, Germany), which is used to construct the underwater acoustic communication network, obtain the relative range measurements between the master and slave AUVs and transfer the reference information. The real-time GPS position information of two master AUVs is used as a reference during the test. The position deduced from the dead reckoning (DR) of the slave AUV is based on the absolute speed information provided by the DVL and the heading information provided by the magnetic compass with a 1 Hz frequency. At the same time, a GPS/Photonics Inertial Navigation System (PHINS) integrated navigation system is also installed on the slave AUV to provide relatively accurate position, speed and heading references as benchmarks. The relevant sensor indicators used during the test are shown in Table 1. The slave AUV can only receive one acoustic measurement from the master AUV at a time. Due to the complicated underwater environment, the underwater acoustic measurement information may be lost or garbled. For the convenience of data processing, the received acoustic communication data will be stored only if the measurement information received from the master AUVs is correct.

**Figure 4:**
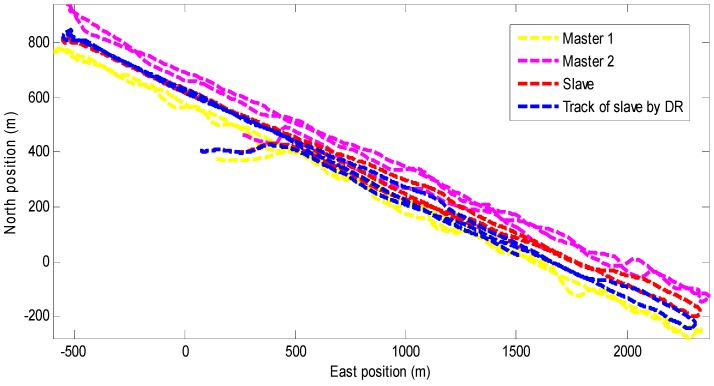
Paths taken by the two master Autonomous Underwater Vehicles (AUVs) and one slave AUV.

The trajectories of the two master AUVs and one slave AUV together with the dead reckoning of slave AUV are drawn in Figure 4. It can be seen that the DR trajectory of the slave vehicle (blue dashed line) gradually diverges from the true trajectory (red dashed line) over time, which will cause a large navigation error.

We use the multi-AUVs cooperative navigation scenario through this lake trial experiment to verify the effectiveness and superiority of the algorithm proposed in this paper. The traditional EKF-based cooperative navigation algorithm and the variational Bayesian-based cooperative navigation algorithm proposed in this paper are both used to estimate the location of the slave AUV and compare their performance. In addition, the Sage-Husa Extended Kalman Filter (SHEKF) and Maximum Likelihood Extended Kalman Filter (MLEKF) are also introduced to verify the superiority of the proposed navigation algorithm [31]. They are common methods for estimating unknown noise parameters in practical applications.

The initial state estimate ${\hat{X}}_{0|0}$ is provided by GPS. And the initial state estimation error covariance is set as $P_{0|0}=diag\left[{\left(1m\right)}^{2},{\left(1m\right)}^{2}\right]$. The parameter values of the proposed algorithm and existing algorithms are shown in Table 2. They were carried out in MATLAB R2014 on a computer with an Intel® Core™ i5-3470 CPU 3.20 GHz and 4 GB of RAM. In order to compare the accuracy of the state estimation of the above nonlinear filtering algorithm, the position error and the average position error are chosen as performance indicators, which are defined as follows:

(54) $$\mathrm{Position}\;\mathrm{Error}(k)=\sqrt{{\left(x_{k}-{\hat{x}}_{k|k}\right)}^{2}+{\left(y_{k}-{\hat{y}}_{k|k}\right)}^{2}},$$

(55) $$\mathrm{RMSE}=\frac{1}{T}\sum_{k=1}^{T}\sqrt{{\left(x_{k}-{\hat{x}}_{k}\right)}^{2}+{\left(y_{k}-{\hat{y}}_{k}\right)}^{2}},$$

where $\left(x_{k},y_{k}\right)$ is the reference position of the slave AUV provided by GPS, $\left({\hat{x}}_{k|k},{\hat{y}}_{k|k}\right)$ is the estimated position at time k, and T is the time length.

In order to better demonstrate the effectiveness and superiority of the algorithm, we consider two cases:

**Case 1:** In the first case, we assume that the nominal state noise covariance and measurement noise covariance are constant in EKF, and they are used as the initial values in the proposed VB-based adaptive filter. Then $\bar{Q}$ and $\bar{R}$ are chosen as

(56) $$\bar{Q}=\left[\begin{array}{cc} {\left(0.5m\right)}^{2} & 0\\ 0 & {\left(0.5m\right)}^{2} \end{array}\right]\;\bar{R}={\left(\sqrt{2}m\right)}^{2}.$$

**Figure 5:**
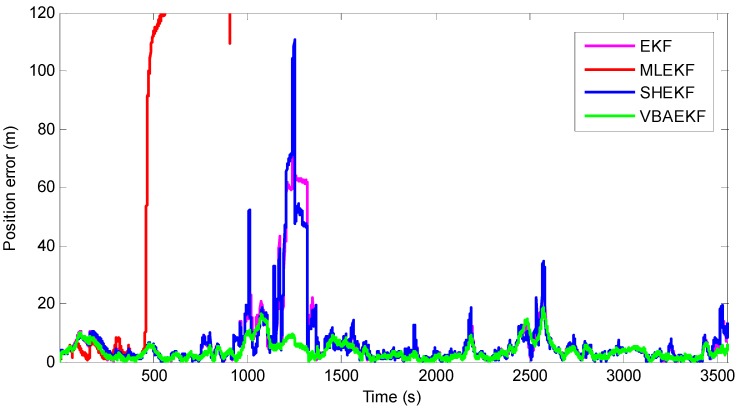
Position estimation errors for Case 1 for the EKF, MLEKF, SHEKF and VBAEKF.

**Figure 6:**
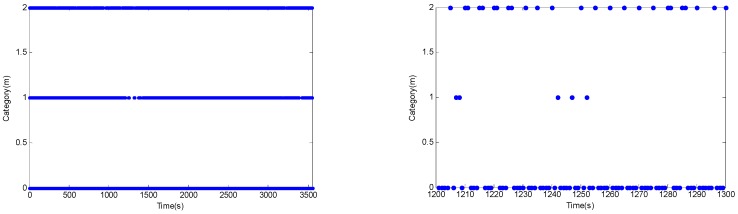
Illustration of the measurements received from the two master AUVs.

The estimated errors from the slave AUV’s position are plotted in Figure 5. The results show that in the traditional EKF-based cooperative navigation algorithm, the state process noise error and measurement noise error do not obey the constant values, resulting in obvious deviation and a long period of time to regain the correct estimate position. In SHEKF, the performance of SHEKF is not ideal because the online estimation of noises is not accurate. From Figure 6, it can be seen that after 1100 s, the drastic decrease in the measured value leads to a large positioning error. The estimated noise covariance matrix of MLEKF is not accurate when an abnormal innovation occurs because the MLEKF relies heavily on the changes of new innovations, which may lead to divergence for the estimated solution. Compared with the traditional EKF, the Sage–Husa adaptive filter and the maximum likelihood adaptive filter, the proposed VB-based adaptive filter can quickly adjust and converge after the position estimation error peak.

Table 3 shows the average positioning error and average execution time for the existing algorithm and the proposed algorithm. It can be seen that the execution time of the traditional EKF and Sage–Husa adaptive algorithm is almost the same, and the implementation time of the proposed VB-based adaptive algorithm is slightly longer than the traditional EKF, taking into account the time of the iteration is reasonable. For many practical applications, this increase in execution time is negligible considering the increase in accuracy.

**Case 2:** In the second case, $\bar{Q}$ and $\bar{R}$ are chosen as large value noises, which are set as:

(57) $$\bar{Q}=\left[\begin{array}{cc} {\left(1\right)}^{2} & 0\\ 0 & {\left(1\right)}^{2} \end{array}\right]\;\bar{R}={\left(\sqrt{30}\right)}^{2},$$

where T=3554 s denotes the test time, and k is the time step.

**Figure 7:**
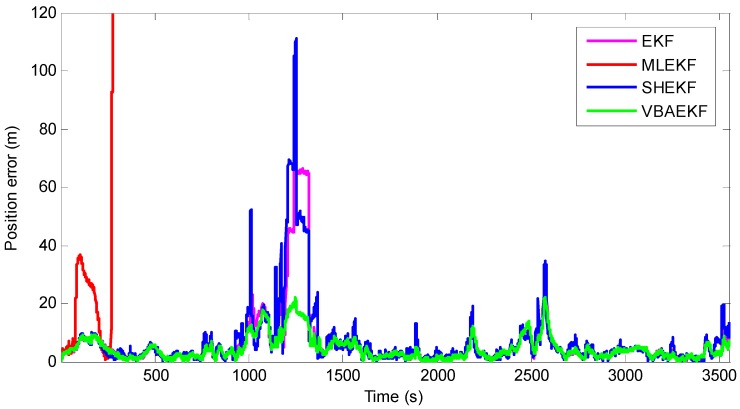
Position estimation errors for Case 2.

Figure 7 shows the position estimation errors of the existing algorithm and the proposed algorithm. It can be seen that when SHEKF processes large value noise parameters, the estimation error is worse than EKF. The performance of MLEKF in the second case diverges faster than in the first case. From Table 4, it can be seen that the average positioning error of the traditional EKF-based cooperative navigation algorithm is 6.33 m. The use of the VBAEKF-based cooperative navigation algorithm reduces the average positioning error to 4.5 m, and the positioning accuracy is improved by 28.9%. The algorithm presented in this paper has obvious improvement in positioning accuracy compared with the existing algorithms. This is because the proposed algorithm can better estimate the prediction covariance matrix and the measurement noise covariance matrix than the existing algorithms. Therefore, compared with other existing cooperative navigation algorithms, the proposed algorithm is more robust against large values of the process noise covariance matrix and measurement noise covariance matrix.

## 5. Conclusions

This paper presents a variational Bayesian adaptive extended Kalman filter algorithm for cooperative navigation of master–slave AUVs. The predicted error covariance matrix and measurement noise covariance matrix are modeled as inverse Wishart priors and are inferred by the VB method together with the system state. In the proposed method, the predicted error covariance matrix is estimated instead of process noise parameters, so the state estimation considers not only the change of noise, but also the variation of the predicted covariance to better model the cooperative navigation of AUVs. It is compared with the typical cooperative navigation algorithms using real experimental data. Experimental results show that the proposed adaptive EKF algorithm is superior to the traditional EKF algorithm and the Sage-Husa adaptive algorithm in terms of positioning error and is suitable for application in multi-AUVs cooperative navigation.

## Figures and Tables

**Table 1:** The performance of the sensors used in experiments.

Sensors	Index	Parameters
S2CR 7/17 (Master/Slave)	Working range	Up to 8000 m
	Data transfer rate	Up to 6.9 kbit/s
	Error rate	Less than $10^{-10}$
GPS (Master/Slave)	Velocity accuracy	0.1 m/s
	Position accuracy	Less than 2.5 m (Root Mean Square (RMS))
	Data update rate	10 Hz
Compass (Slave)	Heading accuracy	2°
DVL (Slave)	Velocity accuracy	0.1%

**Table 2:** Parameter values of the proposed algorithm and existing algorithms.

Filters	Parameter	Value
SHEKF	Forgetting factor	1−exp(4)
MLEKF	Sliding window size	20
The proposed VBAEKF	Forgetting factor	ρ=1−exp(4)
	Tuning parameter	τ=2
	The iteration number	N=5

SHEKF: Sage-Husa Extended Kalman Filter; MLEKF: Maximum Likelihood Extended Kalman Filter; VBAEKF: Variational Bayesian Adaptive Extended Kalman Filter.

**Table 3:** RMSE and the average execution time during Case 1 for the Extended Kalman filter (EKF), SHEKF and the VBAEKF.

Filters	RMSE (m)	Execution Time (s)
EKF	6.92 m	$3.78\times 10^{-5}$
SHEKF	6.64 m	$3.97\times 10^{-5}$
The proposed VBAEKF	3.83 m	$1.38\times 10^{-4}$

**Table 4:** RMSE and the average execution time for Case 2.

Filters	RMSE (m)	Execution Time (s)
EKF	6.33 m	$3.52\times 10^{-5}$
SHEKF	6.65 m	$3.86\times 10^{-5}$
The proposed VBAEKF	4.50 m	$1.34\times 10^{-4}$

## Appendix A. Derivation of (28)

According to Bayes’ theorem, the joint PDF can be expressed as:

(A1) $$p(X_{k},P_{k|k-1},R_{k},Z_{1:k})=p(Z_{k}|X_{k},R_{k})p(X_{k}|Z_{1:k-1},P_{k|k-1})p(P_{k|k-1}|Z_{1:k-1})p(R_{k}|Z_{1:k-1})p(Z_{1:k-1}).$$

Substituting (6), (7), (13) and (14) into (A1) yields:

(A2) $$\begin{array}{c} p(X_{k},P_{k|k-1},R_{k},Z_{1:k})=N(Z_{k};h(X_{k}),R_{k})N(X_{k};{\hat{X}}_{k|k-1},P_{k|k-1})\\ \times IW(P_{k|k-1};{\hat{t}}_{k|k-1},{\hat{T}}_{k|k-1})IW(R_{k};{\hat{u}}_{k|k-1},{\hat{U}}_{k|k-1})p(Z_{1:k-1}). \end{array}$$

Using Equation (8), logN(A;γ,Σ) is formulated as:

(A3) $$\begin{array}{l} \log\left\{N(A;\gamma ,\Sigma )\right\}=\log\left\{\frac{1}{\sqrt{2\pi }}\;{\left|\Sigma \right|}^{-\frac{1}{2}}\;e^{-\frac{1}{2}{\left(A-\gamma \right)}^{T}\Sigma^{-1}(A-\gamma )}\right\}\\ =\log\frac{1}{\sqrt{2\pi }}-\frac{1}{2}\log\left|\Sigma \right|-\frac{1}{2}{\left(A-\gamma \right)}^{T}\Sigma^{-1}(A-\gamma )=-\frac{1}{2}\log\left|\Sigma \right|-\frac{1}{2}{\left(A-\gamma \right)}^{T}\Sigma^{-1}(A-\gamma )+c_{A}, \end{array}$$

where $c_{A}$ represents the constant with respect to variable A.

Using Equation (11), logIW(B;λ,Ψ) is formulated as:

(A4) $$\begin{array}{l} \log\left\{IW(B;\lambda ,\Psi )\right\}\\ =\log\left\{\frac{{\left|\Psi \right|}^{\lambda /2}{\left|B\right|}^{-(\lambda +d+1)/2}e^{-trace(\Psi B^{-1})/2}}{2^{d\lambda /2}\Gamma_{d}(\lambda /2)}\right\}\\ =\frac{\lambda }{2}\log\left|\Psi \right|-\frac{\left(\lambda +d+1\right)}{2}\log\left|B\right|-\frac{1}{2}trace(\Psi B^{-1})-\frac{d\lambda }{2}\log2-\log\Gamma_{d}(\lambda /2)\\ =-\frac{\left(\lambda +d+1\right)}{2}\log\left|B\right|-\frac{1}{2}trace(\Psi B^{-1})+c_{B}, \end{array}$$

where $c_{B}$ represents the constant with respect to variable B.

Using (A2)–(A4), the logarithm of joint PDF can be formulated as:

(A5) $$\begin{array}{l} \log p(X_{k},P_{k|k-1},R_{k},Z_{1:k})\\ =\log\{N(Z_{k};h(X_{k}),R_{k})N(X_{k};{\hat{X}}_{k|k-1},P_{k|k-1})\\ \;\times IW(P_{k|k-1};{\hat{t}}_{k|k-1},{\hat{T}}_{k|k-1})IW(R_{k};{\hat{u}}_{k|k-1},{\hat{U}}_{k|k-1})p(Z_{1:k-1})\\ =-\frac{1}{2}\log\left|R_{k}\right|-\frac{1}{2}{\left(Z_{k}-h(X_{k})\right)}^{T}R_{k}^{-1}\left(Z_{k}-h(X_{k})\right)-\frac{1}{2}\log\left|P_{k|k-1}\right|\\ \;-\frac{1}{2}{\left(X_{k}-{\hat{X}}_{k|k-1}\right)}^{T}P_{k|k-1}^{-1}\left(X_{k}-{\hat{X}}_{k|k-1}\right)-\frac{{\hat{t}}_{k|k-1}+n+1}{2}\log\left|P_{k|k-1}\right|\\ \;-\frac{1}{2}trace\left({\hat{T}}_{k|k-1}P_{k|k-1}^{-1}\right)-\frac{{\hat{u}}_{k|k-1}+m+1}{2}\log\left|R_{k}\right|-\frac{1}{2}trace\left({\hat{U}}_{k|k-1}R_{k}^{-1}\right)+c_{\Theta }\\ =-\frac{1}{2}\left(m+{\hat{u}}_{k|k-1}+2\right)\log\left|R_{k}\right|-\frac{1}{2}{\left(Z_{k}-h(X_{k})\right)}^{T}R_{k}^{-1}\left(Z_{k}-h(X_{k})\right)\\ \;-\frac{1}{2}trace\left({\hat{U}}_{k|k-1}R_{k}^{-1}\right)-\frac{1}{2}(n+{\hat{t}}_{k|k-1}+2)\log\left|P_{k|k-1}\right|\\ \;-\frac{1}{2}{\left(X_{k}-{\hat{X}}_{k|k-1}\right)}^{T}P_{k|k-1}^{-1}(X_{k}-{\hat{X}}_{k|k-1})-\frac{1}{2}trace\left({\hat{T}}_{k|k-1}P_{k|k-1}^{-1}\right)+c_{\Theta }, \end{array}$$

where $c_{\Theta }$ represents the constant with respect to variables $X_{k},P_{k|k-1}$ and $R_{k}$.

Using (A5) in (26), the detailed derivation of Equation (28) is presented as follows:

(A6) $$\begin{array}{l} \log q^{\left(i+1\right)}(P_{k|k-1})=E_{X_{k},R_{k}}^{\left(i\right)}\left[\log p(X_{k},P_{k|k-1},R_{k},Z_{1:k})\right]+c_{P_{k|k-1}}\\ =-\frac{1}{2}\left(m+{\hat{u}}_{k|k-1}+2\right)E^{\left(i\right)}\left[\log\left|R_{k}\right|\right]-\frac{1}{2}E^{\left(i\right)}\left[{\left(Z_{k}-h(X_{k})\right)}^{T}R_{k}^{-1}\left(Z_{k}-h(X_{k})\right)\right]\\ \;-\frac{1}{2}E^{\left(i\right)}\left[trace\left({\hat{U}}_{k|k-1}R_{k}^{-1}\right)\right]-\frac{1}{2}(n+{\hat{t}}_{k|k-1}+2)\log\left|P_{k|k-1}\right|\\ \;-\frac{1}{2}trace\left(A_{k}^{\left(i\right)}+{\hat{T}}_{k|k-1}P_{k|k-1}^{-1}\right)+c_{\Theta }\\ =-\frac{1}{2}(n+{\hat{t}}_{k|k-1}+2)\log\left|P_{k|k-1}\right|-\frac{1}{2}trace\left(\left(A_{k}^{\left(i\right)}+{\hat{T}}_{k|k-1}\right)P_{k|k-1}^{-1}\right)+c_{P_{k|k-1}}, \end{array}$$

where

$$\begin{array}{cc} c_{P_{k|k-1}} & =-\frac{1}{2}\left(m+{\hat{u}}_{k|k-1}+2\right)E^{\left(i\right)}\left[\log\left|R_{k}\right|\right]-\frac{1}{2}E^{\left(i\right)}\left[{\left(Z_{k}-h(X_{k})\right)}^{T}R_{k}^{-1}\left(Z_{k}-h(X_{k})\right)\right]\\ & \;-\frac{1}{2}E^{\left(i\right)}\left[trace\left({\hat{U}}_{k|k-1}R_{k}^{-1}\right)\right]+c_{\Theta }. \end{array}$$

Similarly, Equations (33) and (38) can also be obtained.

## References

[1] Leonard J.J., Bahr A. (2016). Autonomous Underwater Vehicle Navigation.

[2] Paull L., Saeedi S., Seto M., Li H. (2014). AUV Navigation and Localization: A Review. IEEE J. Ocean. Eng..

[3] Fallon M.F., Papadopoulos G., Leonard J.J. A measurement distribution framework for cooperative navigation using multiple AUVs. Proceedings of the 2010 IEEE International Conference on Robotics and Automation (ICRA).

[4] Tsiogkas N., Saigol Z., Lane D. Distributed Multi-AUV Cooperation Methods for Underwater Archaeology. Proceedings of the OCEANS 2015.

[5] Paull L., Seto M., Leonard J.J. Decentralized cooperative trajectory estimation for autonomous underwater vehicles. Proceedings of the 2014 IEEE/RSJ International Conference on Intelligent Robots and Systems (IROS 2014).

[6] Munafò A., Ferri G. (2017). An Acoustic Network Navigation System. J. Field Robot..

[7] Huang Y.L., Zhang Y.G., Xu B., Wu Z.M., Chambers J. (2017). A New Outlier-Robust Student’s t Based Gaussian Approximate Filter for Cooperative Localization. IEEE/ASME Trans. Mech..

[8] Stojanovic M. (2007). On the relationship between capacity and distance in an underwater acoustic communication channel. ACM Sigmob. Mob. Comput. Commun. Rev..

[9] Caiti A., Calabrò V., Fabbri T., Fenucci D., Munafò A. Underwater communication and distributed localization of AUV teams. Proceedings of the 2013 MTS/IEEE OCEANS.

[10] Leonard N.E., Paley D.A., Lekien F., Sepulchre R., Fratantoni D.M., Davis R.E. (2007). Collective Motion, Sensor Networks, and Ocean Sampling. Proc. IEEE.

[11] Bahr A., Leonard J.J., Fallon M.F. (2009). Cooperative Localization for Autonomous Underwater Vehicles. Int. J. Robot. Res..

[12] Huang Y., Zhang Y., Wang X. (2017). Kalman-Filtering-Based In-Motion Coarse Alignment for Odometer-Aided SINS. IEEE Trans. Instrum. Meas..

[13] Zhang Y., Huang Y., Li N., Zhao L. (2015). Embedded cubature Kalman filter with adaptive setting of free parameter. Signal Process..

[14] Zhang Y., Huang Y., Li N., Zhao L. (2015). Interpolatory cubature Kalman filters. Control Theory Appl. IET.

[15] Partan J., Kurose J., Levine B.N. (2007). A survey of practical issues in underwater networks. ACM Sigmob. Mob. Comput. Commun. Rev..

[16] Mohammad G.S., Alireza S., Tuleen B. (2016). A Novel Cooperative Opportunistic Routing Scheme for Underwater Sensor Networks. Sensors.

[17] Huang Y., Zhang Y., Wu Z., Li N., Chambers J. (2017). A Novel Adaptive Kalman Filter with Inaccurate Process and Measurement Noise Covariance Matrices. IEEE Trans. Autom. Control.

[18] Narasimhappa M., Mahindrakar A.D., Guizilini V.C., Terra M.H., Sabat S.L. An improved Sage Husa adaptive robust Kalman Filter for de-noising the MEMS IMU drift signal. Proceedings of the Indian Control Conference.

[19] Huang Y., Zhang Y. (2017). Robust Student’s t-Based Stochastic Cubature Filter for Nonlinear Systems with Heavy-Tailed Process and Measurement Noises. IEEE Access.

[20] Huang Y., Zhang Y. (2017). A New Process Uncertainty Robust Student’s t based Kalman Filter for SINS/GPS Integration. IEEE Access.

[21] Hajiyev C., Vural S.Y., Hajiyeva U. Adaptive Fading Kalman Filter with Q-adaptation for estimation of AUV dynamics. Proceedings of the Control & Automation.

[22] Nagaraju V., Fiondella L., Zeephongsekul P., Wandji T. (2017). An Adaptive EM Algorithm for the Maximum Likelihood Estimation of Non-Homogeneous Poisson Process Software Reliability Growth Models. Int. J. Reliab. Qual. Saf. Eng..

[23] Wang G., Li N., Zhang Y. (2017). Maximum correntropy unscented Kalman and information filters for non-Gaussian measurement noise. J. Frankl. Inst..

[24] Wang G., Li N., Zhang Y. (2017). Diffusion nonlinear Kalman filter with intermittent observations. Proc. Inst. Mech. Eng. Part G.

[25] Huang Y., Zhang Y., Li N., Zhao L. (2016). Design of Sigma-Point Kalman Filter with Recursive Updated Measurement. Circuits Syst. Signal Process..

[26] Fallon M.F., Papadopoulos G., Leonard J.J. (2010). Cooperative AUV Navigation Using a Single Surface Craft. Field and Service Robotics.

[27] Lindley D. (2005). Kendall’s Advanced Theory of Statistics, volume 2B, Bayesian Inference. J. R. Stat. Soc..

[28] Hartikainen S.S.J. (2013). Variational Bayesian Adaptation of Noise Covariances in Non-Linear Kalman Filtering. arXiv.

[29] Tzikas D.G., Likas C.L., Galatsanos N.P. (2008). The variational approximation for Bayesian inference. Signal Process. Mag..

[30] Bishop C.M. (2006). Pattern Recognition and Machine Learning.

[31] Narasimhappa M., Rangababu P., Sabat S.L., Nayak J. In A modified Sage-Husa adaptive Kalman filter for denoising fiber optic gyroscope signal. Proceedings of the India Conference (INDICON).

